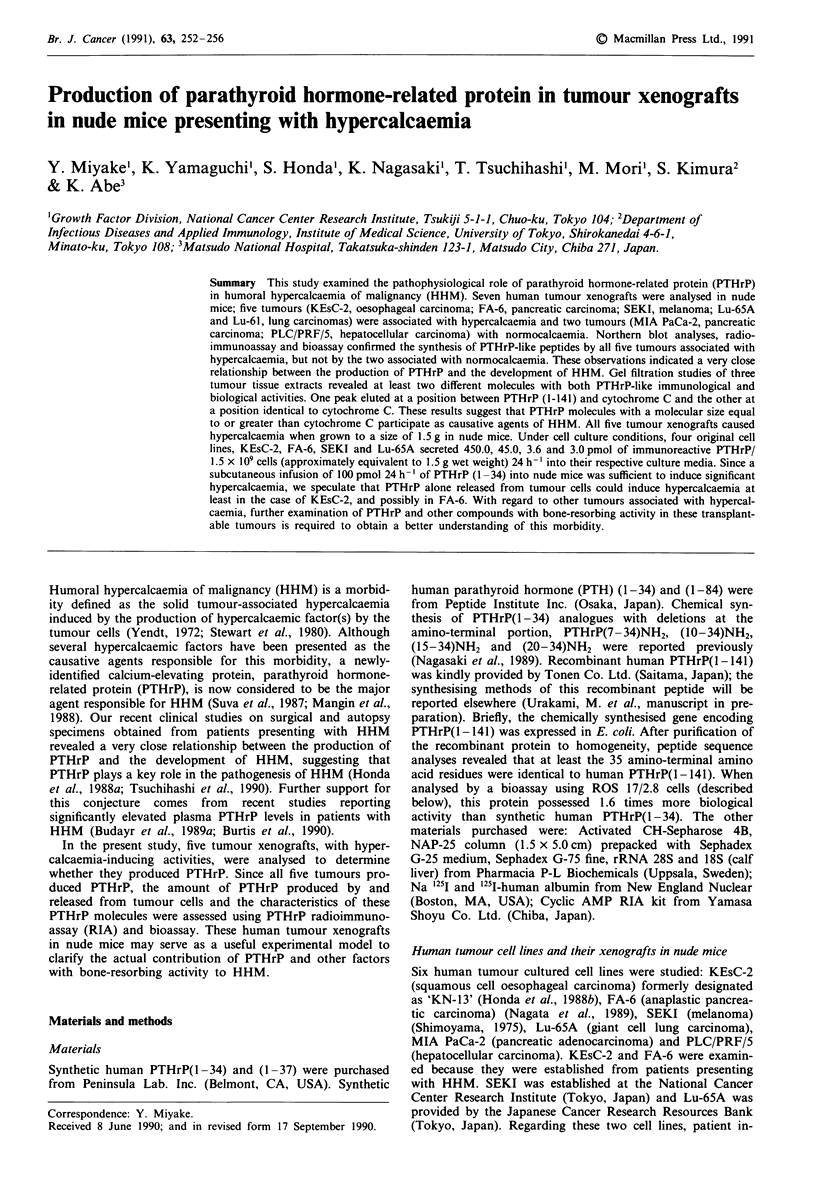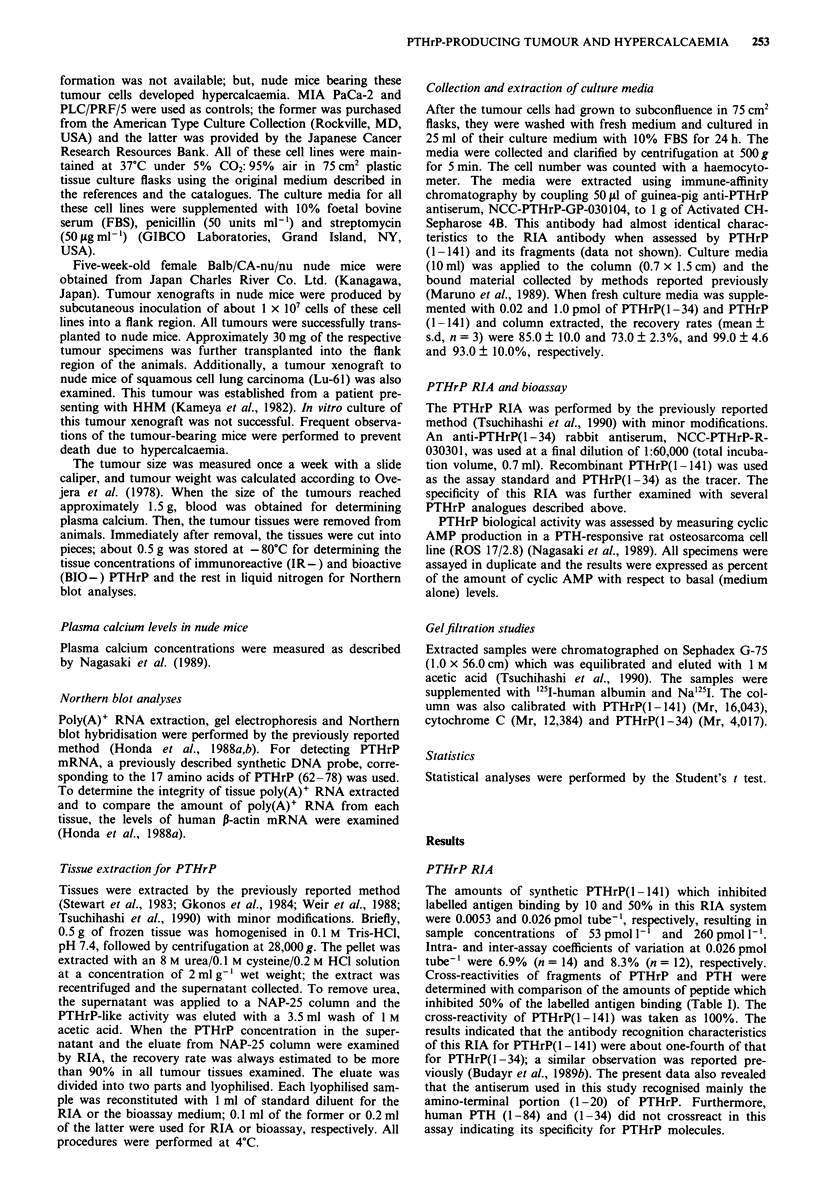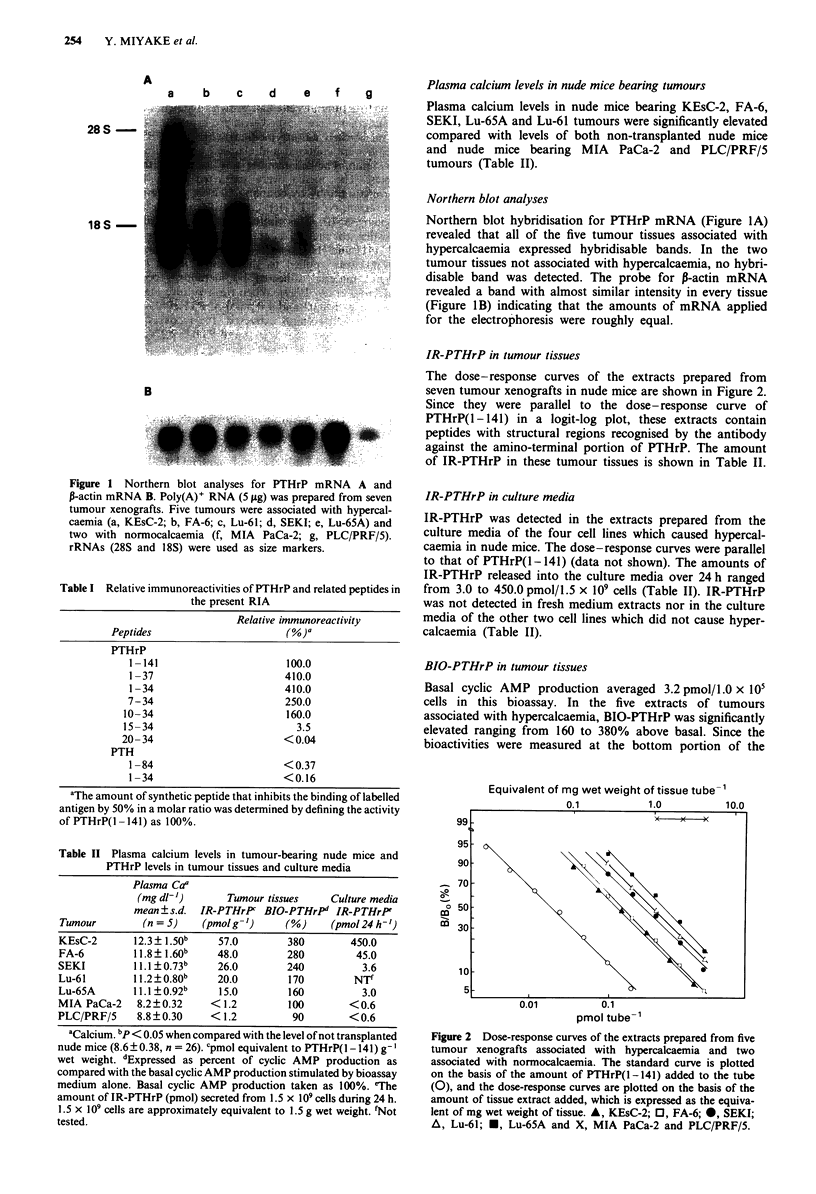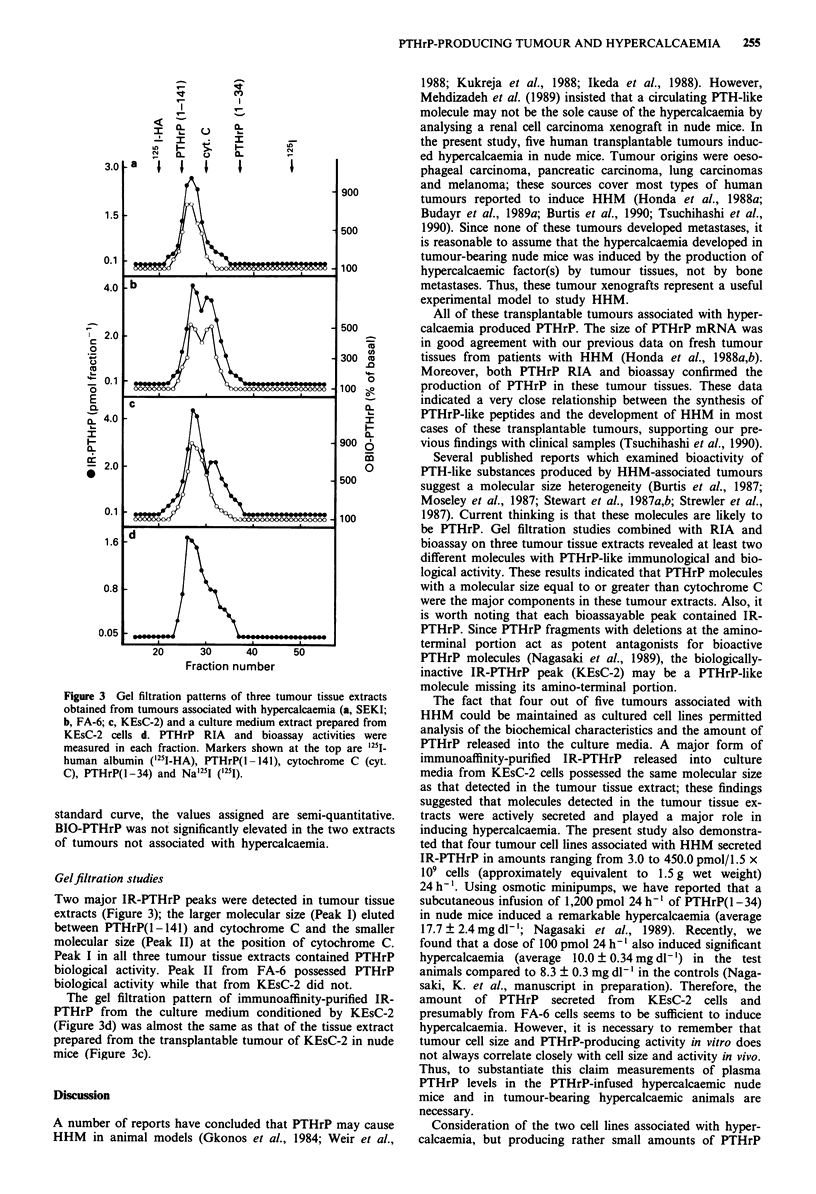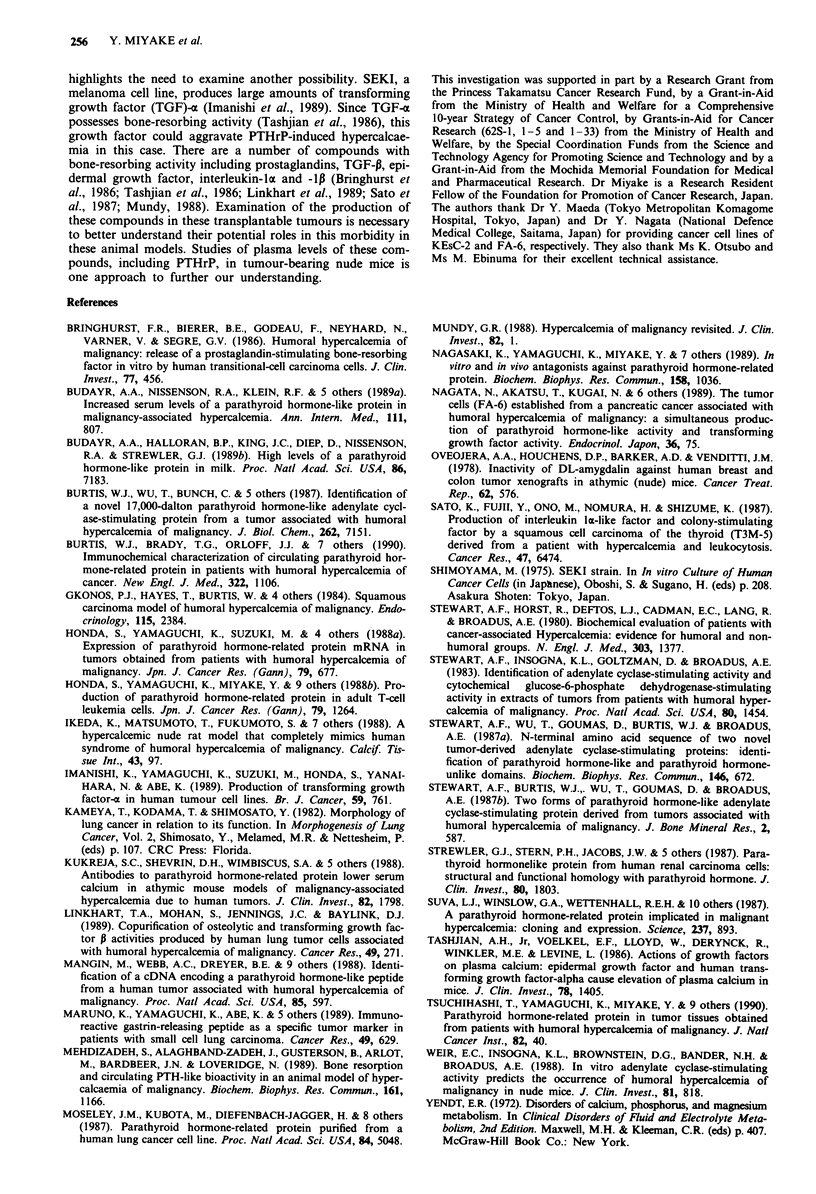# Production of parathyroid hormone-related protein in tumour xenografts in nude mice presenting with hypercalcaemia.

**DOI:** 10.1038/bjc.1991.59

**Published:** 1991-02

**Authors:** Y. Miyake, K. Yamaguchi, S. Honda, K. Nagasaki, T. Tsuchihashi, M. Mori, S. Kimura, K. Abe

**Affiliations:** Growth Factor Division, National Cancer Center Research Institute, Tokyo, Japan.

## Abstract

**Images:**


*Br. J. Cancer* (1991), **63**, 252–256 © Macmillan Press Ltd., 1991

### Production of parathyroid hormone-related protein in tumour xenografts in nude mice presenting with hypercalcaemia

Y. Miyake[1], K. Yamaguchi[1], S. Honda[1], K. Nagasaki[1], T. Tsuchihashi[1], M. Mori[1], S. Kimura[2] & K. Abe[3]

[1]*Growth Factor Division, National Cancer Center Research Institute, Tsukiji 5-1-1, Chuo-ku, Tokyo 104;* [2]*Department of Infectious Diseases and Applied Immunology, Institute of Medical Science, University of Tokyo, Shirokanedai 4-6-1, Minato-ku, Tokyo 108;* [3]*Matsudo National Hospital, Takatsuka-shinden 123-1, Matsudo City, Chiba 271, Japan.*

**Summary** This study examined the pathophysiological role of parathyroid hormone-related protein (PTHrP) in humoral hypercalcaemia of malignancy (HHM). Seven human tumour xenografts were analysed in nude mice; five tumours (KEsC-2, oesophageal carcinoma; FA-6, pancreatic carcinoma; SEKI, melanoma; Lu-65A and Lu-61, lung carcinomas) were associated with hypercalcaemia and two tumours (MIA PaCa-2, pancreatic carcinoma; PLC/PRF/5, hepatocellular carcinoma) with normocalcaemia. Northern blot analyses, radioimmunoassay and bioassay confirmed the synthesis of PTHrP-like peptides by all five tumours associated with hypercalcaemia, but not by the two associated with normocalcaemia. These observations indicated a very close relationship between the production of PTHrP and the development of HHM. Gel filtration studies of three tumour tissue extracts revealed at least two different molecules with both PTHrP-like immunological and biological activities. One peak eluted at a position between PTHrP (1-141) and cytochrome C and the other at a position identical to cytochrome C. These results suggest that PTHrP molecules with a molecular size equal to or greater than cytochrome C participate as causative agents of HHM. All five tumour xenografts caused hypercalcaemia when grown to a size of 1.5 g in nude mice. Under cell culture conditions, four original cell lines, KEsC-2, FA-6, SEKI and Lu-65A secreted 450.0, 45.0, 3.6 and 3.0 pmol of immunoreactive PTHrP/ $1.5 \times 10^9$ cells (approximately equivalent to 1.5 g wet weight) 24 $h^{-1}$ into their respective culture media. Since a subcutaneous infusion of 100 pmol 24 $h^{-1}$ of PTHrP (1–34) into nude mice was sufficient to induce significant hypercalcaemia, we speculate that PTHrP alone released from tumour cells could induce hypercalcaemia at least in the case of KEsC-2, and possibly in FA-6. With regard to other tumours associated with hypercalcaemia, further examination of PTHrP and other compounds with bone-resorbing activity in these transplantable tumours is required to obtain a better understanding of this morbidity.

Humoral hypercalcaemia of malignancy (HHM) is a morbidity defined as the solid tumour-associated hypercalcaemia induced by the production of hypercalcaemic factor(s) by the tumour cells (Yendt, 1972; Stewart *et al.*, 1980). Although several hypercalcaemic factors have been presented as the causative agents responsible for this morbidity, a newly-identified calcium-elevating protein, parathyroid hormone-related protein (PTHrP), is now considered to be the major agent responsible for HHM (Suva *et al.*, 1987; Mangin *et al.*, 1988). Our recent clinical studies on surgical and autopsy specimens obtained from patients presenting with HHM revealed a very close relationship between the production of PTHrP and the development of HHM, suggesting that PTHrP plays a key role in the pathogenesis of HHM (Honda *et al.*, 1988*a*; Tsuchihashi *et al.*, 1990). Further support for this conjecture comes from recent studies reporting significantly elevated plasma PTHrP levels in patients with HHM (Budayr *et al.*, 1989*a*; Burtis *et al.*, 1990).

In the present study, five tumour xenografts, with hypercalcaemia-inducing activities, were analysed to determine whether they produced PTHrP. Since all five tumours produced PTHrP, the amount of PTHrP produced by and released from tumour cells and the characteristics of these PTHrP molecules were assessed using PTHrP radioimmunoassay (RIA) and bioassay. These human tumour xenografts in nude mice may serve as a useful experimental model to clarify the actual contribution of PTHrP and other factors with bone-resorbing activity to HHM.

### Materials and methods

### *Materials*

Synthetic human PTHrP(1–34) and (1–37) were purchased from Peninsula Lab. Inc. (Belmont, CA, USA). Synthetic human parathyroid hormone (PTH) (1–34) and (1–84) were from Peptide Institute Inc. (Osaka, Japan). Chemical synthesis of PTHrP(1–34) analogues with deletions at the amino-terminal portion, PTHrP(7–34)$NH_2$, (10–34)$NH_2$, (15–34)$NH_2$ and (20–34)$NH_2$ were reported previously (Nagasaki *et al.*, 1989). Recombinant human PTHrP(1–141) was kindly provided by Tonen Co. Ltd. (Saitama, Japan); the synthesising methods of this recombinant peptide will be reported elsewhere (Urakami, M. *et al.*, manuscript in preparation). Briefly, the chemically synthesised gene encoding PTHrP(1–141) was expressed in *E. coli*. After purification of the recombinant protein to homogeneity, peptide sequence analyses revealed that at least the 35 amino-terminal amino acid residues were identical to human PTHrP(1–141). When analysed by a bioassay using ROS 17/2.8 cells (described below), this protein possessed 1.6 times more biological activity than synthetic human PTHrP(1–34). The other materials purchased were: Activated CH-Sepharose 4B, NAP-25 column (1.5 × 5.0 cm) prepacked with Sephadex G-25 medium, Sephadex G-75 fine, rRNA 28S and 18S (calf liver) from Pharmacia P-L Biochemicals (Uppsala, Sweden); Na $^{125}I$ and $^{125}I$-human albumin from New England Nuclear (Boston, MA, USA); Cyclic AMP RIA kit from Yamasa Shoyu Co. Ltd. (Chiba, Japan).

### *Human tumour cell lines and their xenografts in nude mice*

Six human tumour cultured cell lines were studied: KEsC-2 (squamous cell oesophageal carcinoma) formerly designated as 'KN-13' (Honda *et al.*, 1988*b*), FA-6 (anaplastic pancreatic carcinoma) (Nagata *et al.*, 1989), SEKI (melanoma) (Shimoyama, 1975), Lu-65A (giant cell lung carcinoma), MIA PaCa-2 (pancreatic adenocarcinoma) and PLC/PRF/5 (hepatocellular carcinoma). KEsC-2 and FA-6 were examined because they were established from patients presenting with HHM. SEKI was established at the National Cancer Center Research Institute (Tokyo, Japan) and Lu-65A was provided by the Japanese Cancer Research Resources Bank (Tokyo, Japan). Regarding these two cell lines, patient in-

Correspondence: Y. Miyake.
Received 8 June 1990; and in revised form 17 September 1990.

formation was not available; but, nude mice bearing these tumour cells developed hypercalcaemia. MIA PaCa-2 and PLC/PRF/5 were used as controls; the former was purchased from the American Type Culture Collection (Rockville, MD, USA) and the latter was provided by the Japanese Cancer Research Resources Bank. All of these cell lines were maintained at 37°C under 5% $CO_2$: 95% air in 75 $cm^2$ plastic tissue culture flasks using the original medium described in the references and the catalogues. The culture media for all these cell lines were supplemented with 10% foetal bovine serum (FBS), penicillin (50 units $ml^{-1}$) and streptomycin (50 $\mu g\ ml^{-1}$) (GIBCO Laboratories, Grand Island, NY, USA).

Five-week-old female Balb/CA-nu/nu nude mice were obtained from Japan Charles River Co. Ltd. (Kanagawa, Japan). Tumour xenografts in nude mice were produced by subcutaneous inoculation of about $1 \times 10^7$ cells of these cell lines into a flank region. All tumours were successfully transplanted to nude mice. Approximately 30 mg of the respective tumour specimens was further transplanted into the flank region of the animals. Additionally, a tumour xenograft to nude mice of squamous cell lung carcinoma (Lu-61) was also examined. This tumour was established from a patient presenting with HHM (Kameya *et al.*, 1982). *In vitro* culture of this tumour xenograft was not successful. Frequent observations of the tumour-bearing mice were performed to prevent death due to hypercalcaemia.

The tumour size was measured once a week with a slide caliper, and tumour weight was calculated according to Ovejera *et al.* (1978). When the size of the tumours reached approximately 1.5 g, blood was obtained for determining plasma calcium. Then, the tumour tissues were removed from animals. Immediately after removal, the tissues were cut into pieces; about 0.5 g was stored at −80°C for determining the tissue concentrations of immunoreactive (IR−) and bioactive (BIO−) PTHrP and the rest in liquid nitrogen for Northern blot analyses.

*Plasma calcium levels in nude mice*

Plasma calcium concentrations were measured as described by Nagasaki *et al.* (1989).

*Northern blot analyses*

Poly(A)$^+$ RNA extraction, gel electrophoresis and Northern blot hybridisation were performed by the previously reported method (Honda *et al.*, 1988*a,b*). For detecting PTHrP mRNA, a previously described synthetic DNA probe, corresponding to the 17 amino acids of PTHrP (62–78) was used. To determine the integrity of tissue poly(A)$^+$ RNA extracted and to compare the amount of poly(A)$^+$ RNA from each tissue, the levels of human $\beta$-actin mRNA were examined (Honda *et al.*, 1988*a*).

*Tissue extraction for PTHrP*

Tissues were extracted by the previously reported method (Stewart *et al.*, 1983; Gkonos *et al.*, 1984; Weir *et al.*, 1988; Tsuchihashi *et al.*, 1990) with minor modifications. Briefly, 0.5 g of frozen tissue was homogenised in 0.1 M Tris-HCl, pH 7.4, followed by centrifugation at 28,000 *g*. The pellet was extracted with an 8 M urea/0.1 M cysteine/0.2 M HCl solution at a concentration of 2 ml $g^{-1}$ wet weight; the extract was recentrifuged and the supernatant collected. To remove urea, the supernatant was applied to a NAP-25 column and the PTHrP-like activity was eluted with a 3.5 ml wash of 1 M acetic acid. When the PTHrP concentration in the supernatant and the eluate from NAP-25 column were examined by RIA, the recovery rate was always estimated to be more than 90% in all tumour tissues examined. The eluate was divided into two parts and lyophilised. Each lyophilised sample was reconstituted with 1 ml of standard diluent for the RIA or the bioassay medium; 0.1 ml of the former or 0.2 ml of the latter were used for RIA or bioassay, respectively. All procedures were performed at 4°C.

*Collection and extraction of culture media*

After the tumour cells had grown to subconfluence in 75 $cm^2$ flasks, they were washed with fresh medium and cultured in 25 ml of their culture medium with 10% FBS for 24 h. The media were collected and clarified by centrifugation at 500 *g* for 5 min. The cell number was counted with a haemocytometer. The media were extracted using immune-affinity chromatography by coupling 50 $\mu$l of guinea-pig anti-PTHrP antiserum, NCC-PTHrP-GP-030104, to 1 g of Activated CH-Sepharose 4B. This antibody had almost identical characteristics to the RIA antibody when assessed by PTHrP (1–141) and its fragments (data not shown). Culture media (10 ml) was applied to the column (0.7 × 1.5 cm) and the bound material collected by methods reported previously (Maruno *et al.*, 1989). When fresh culture media was supplemented with 0.02 and 1.0 pmol of PTHrP(1–34) and PTHrP (1–141) and column extracted, the recovery rates (mean ± s.d, $n = 3$) were 85.0 ± 10.0 and 73.0 ± 2.3%, and 99.0 ± 4.6 and 93.0 ± 10.0%, respectively.

*PTHrP RIA and bioassay*

The PTHrP RIA was performed by the previously reported method (Tsuchihashi *et al.*, 1990) with minor modifications. An anti-PTHrP(1–34) rabbit antiserum, NCC-PTHrP-R-030301, was used at a final dilution of 1:60,000 (total incubation volume, 0.7 ml). Recombinant PTHrP(1–141) was used as the assay standard and PTHrP(1–34) as the tracer. The specificity of this RIA was further examined with several PTHrP analogues described above.

PTHrP biological activity was assessed by measuring cyclic AMP production in a PTH-responsive rat osteosarcoma cell line (ROS 17/2.8) (Nagasaki *et al.*, 1989). All specimens were assayed in duplicate and the results were expressed as percent of the amount of cyclic AMP with respect to basal (medium alone) levels.

*Gel filtration studies*

Extracted samples were chromatographed on Sephadex G-75 (1.0 × 56.0 cm) which was equilibrated and eluted with 1 M acetic acid (Tsuchihashi *et al.*, 1990). The samples were supplemented with $^{125}$I-human albumin and Na$^{125}$I. The column was also calibrated with PTHrP(1–141) (Mr, 16,043), cytochrome C (Mr, 12,384) and PTHrP(1–34) (Mr, 4,017).

*Statistics*

Statistical analyses were performed by the Student's *t* test.

### Results

*PTHrP RIA*

The amounts of synthetic PTHrP(1–141) which inhibited labelled antigen binding by 10 and 50% in this RIA system were 0.0053 and 0.026 pmol $tube^{-1}$, respectively, resulting in sample concentrations of 53 pmol $l^{-1}$ and 260 pmol $l^{-1}$. Intra- and inter-assay coefficients of variation at 0.026 pmol $tube^{-1}$ were 6.9% ($n = 14$) and 8.3% ($n = 12$), respectively. Cross-reactivities of fragments of PTHrP and PTH were determined with comparison of the amounts of peptide which inhibited 50% of the labelled antigen binding (Table I). The cross-reactivity of PTHrP(1–141) was taken as 100%. The results indicated that the antibody recognition characteristics of this RIA for PTHrP(1–141) were about one-fourth of that for PTHrP(1–34); a similar observation was reported previously (Budayr *et al.*, 1989*b*). The present data also revealed that the antiserum used in this study recognised mainly the amino-terminal portion (1–20) of PTHrP. Furthermore, human PTH (1–84) and (1–34) did not crossreact in this assay indicating its specificity for PTHrP molecules.

**Figure 1** Northern blot analyses for PTHrP mRNA **A** and β-actin mRNA **B**. Poly(A)$^+$ RNA (5 μg) was prepared from seven tumour xenografts. Five tumours were associated with hypercalcaemia (a, KEsC-2; b, FA-6; c, Lu-61; d, SEKI; e, Lu-65A) and two with normocalcaemia (f, MIA PaCa-2; g, PLC/PRF/5). rRNAs (28S and 18S) were used as size markers.

**Table I** Relative immunoreactivities of PTHrP and related peptides in the present RIA

| *Peptides* | *Relative immunoreactivity (%)*[a] |
|---|---|
| PTHrP | |
| 1–141 | 100.0 |
| 1–37 | 410.0 |
| 1–34 | 410.0 |
| 7–34 | 250.0 |
| 10–34 | 160.0 |
| 15–34 | 3.5 |
| 20–34 | <0.04 |
| PTH | |
| 1–84 | <0.37 |
| 1–34 | <0.16 |

[a]The amount of synthetic peptide that inhibits the binding of labelled antigen by 50% in a molar ratio was determined by defining the activity of PTHrP(1–141) as 100%.

**Table II** Plasma calcium levels in tumour-bearing nude mice and PTHrP levels in tumour tissues and culture media

| *Tumour* | *Plasma Ca*[a] *(mg dl*$^{-1}$*) mean±s.d. (n = 5)* | *Tumour tissues IR-PTHrP*[c] *(pmol g*$^{-1}$*)* | *Tumour tissues BIO-PTHrP*[d] *(%)* | *Culture media IR-PTHrP*[e] *(pmol 24 h*$^{-1}$*)* |
|---|---|---|---|---|
| KEsC-2 | 12.3±1.50[b] | 57.0 | 380 | 450.0 |
| FA-6 | 11.8±1.60[b] | 48.0 | 280 | 45.0 |
| SEKI | 11.1±0.73[b] | 26.0 | 240 | 3.6 |
| Lu-61 | 11.2±0.80[b] | 20.0 | 170 | NT[f] |
| Lu-65A | 11.1±0.92[b] | 15.0 | 160 | 3.0 |
| MIA PaCa-2 | 8.2±0.32 | <1.2 | 100 | <0.6 |
| PLC/PRF/5 | 8.8±0.30 | <1.2 | 90 | <0.6 |

[a]Calcium. [b]$P < 0.05$ when compared with the level of not transplanted nude mice (8.6±0.38, $n = 26$). [c]pmol equivalent to PTHrP(1–141) g$^{-1}$ wet weight. [d]Expressed as percent of cyclic AMP production as compared with the basal cyclic AMP production stimulated by bioassay medium alone. Basal cyclic AMP production taken as 100%. [e]The amount of IR-PTHrP (pmol) secreted from 1.5 × 10$^9$ cells during 24 h. 1.5 × 10$^9$ cells are approximately equivalent to 1.5 g wet weight. [f]Not tested.

*Plasma calcium levels in nude mice bearing tumours*

Plasma calcium levels in nude mice bearing KEsC-2, FA-6, SEKI, Lu-65A and Lu-61 tumours were significantly elevated compared with levels of both non-transplanted nude mice and nude mice bearing MIA PaCa-2 and PLC/PRF/5 tumours (Table II).

*Northern blot analyses*

Northern blot hybridisation for PTHrP mRNA (Figure 1A) revealed that all of the five tumour tissues associated with hypercalcaemia expressed hybridisable bands. In the two tumour tissues not associated with hypercalcaemia, no hybridisable band was detected. The probe for β-actin mRNA revealed a band with almost similar intensity in every tissue (Figure 1B) indicating that the amounts of mRNA applied for the electrophoresis were roughly equal.

*IR-PTHrP in tumour tissues*

The dose–response curves of the extracts prepared from seven tumour xenografts in nude mice are shown in Figure 2. Since they were parallel to the dose–response curve of PTHrP(1–141) in a logit-log plot, these extracts contain peptides with structural regions recognised by the antibody against the amino-terminal portion of PTHrP. The amount of IR-PTHrP in these tumour tissues is shown in Table II.

*IR-PTHrP in culture media*

IR-PTHrP was detected in the extracts prepared from the culture media of the four cell lines which caused hypercalcaemia in nude mice. The dose–response curves were parallel to that of PTHrP(1–141) (data not shown). The amounts of IR-PTHrP released into the culture media over 24 h ranged from 3.0 to 450.0 pmol/1.5 × 10$^9$ cells (Table II). IR-PTHrP was not detected in fresh medium extracts nor in the culture media of the other two cell lines which did not cause hypercalcaemia (Table II).

*BIO-PTHrP in tumour tissues*

Basal cyclic AMP production averaged 3.2 pmol/1.0 × 10$^5$ cells in this bioassay. In the five extracts of tumours associated with hypercalcaemia, BIO-PTHrP was significantly elevated ranging from 160 to 380% above basal. Since the bioactivities were measured at the bottom portion of the

**Figure 2** Dose-response curves of the extracts prepared from five tumour xenografts associated with hypercalcaemia and two associated with normocalcaemia. The standard curve is plotted on the basis of the amount of PTHrP(1–141) added to the tube (O), and the dose-response curves are plotted on the basis of the amount of tissue extract added, which is expressed as the equivalent of mg wet weight of tissue. ▲, KEsC-2; □, FA-6; ●, SEKI; Δ, Lu-61; ■, Lu-65A and X, MIA PaCa-2 and PLC/PRF/5.

**Figure 3** Gel filtration patterns of three tumour tissue extracts obtained from tumours associated with hypercalcaemia (**a**, SEKI; **b**, FA-6; **c**, KEsC-2) and a culture medium extract prepared from KEsC-2 cells **d**. PTHrP RIA and bioassay activities were measured in each fraction. Markers shown at the top are $^{125}$I-human albumin ($^{125}$I-HA), PTHrP(1–141), cytochrome C (cyt. C), PTHrP(1–34) and Na$^{125}$I ($^{125}$I).

standard curve, the values assigned are semi-quantitative. BIO-PTHrP was not significantly elevated in the two extracts of tumours not associated with hypercalcaemia.

### *Gel filtration studies*

Two major IR-PTHrP peaks were detected in tumour tissue extracts (Figure 3); the larger molecular size (Peak I) eluted between PTHrP(1–141) and cytochrome C and the smaller molecular size (Peak II) at the position of cytochrome C. Peak I in all three tumour tissue extracts contained PTHrP biological activity. Peak II from FA-6 possessed PTHrP biological activity while that from KEsC-2 did not.

The gel filtration pattern of immunoaffinity-purified IR-PTHrP from the culture medium conditioned by KEsC-2 (Figure 3d) was almost the same as that of the tissue extract prepared from the transplantable tumour of KEsC-2 in nude mice (Figure 3c).

### Discussion

A number of reports have concluded that PTHrP may cause HHM in animal models (Gkonos *et al.*, 1984; Weir *et al.*, 1988; Kukreja *et al.*, 1988; Ikeda *et al.*, 1988). However, Mehdizadeh *et al.* (1989) insisted that a circulating PTH-like molecule may not be the sole cause of the hypercalcaemia by analysing a renal cell carcinoma xenograft in nude mice. In the present study, five human transplantable tumours induced hypercalcaemia in nude mice. Tumour origins were oesophageal carcinoma, pancreatic carcinoma, lung carcinomas and melanoma; these sources cover most types of human tumours reported to induce HHM (Honda *et al.*, 1988*a*; Budayr *et al.*, 1989*a*; Burtis *et al.*, 1990; Tsuchihashi *et al.*, 1990). Since none of these tumours developed metastases, it is reasonable to assume that the hypercalcaemia developed in tumour-bearing nude mice was induced by the production of hypercalcaemic factor(s) by tumour tissues, not by bone metastases. Thus, these tumour xenografts represent a useful experimental model to study HHM.

All of these transplantable tumours associated with hypercalcaemia produced PTHrP. The size of PTHrP mRNA was in good agreement with our previous data on fresh tumour tissues from patients with HHM (Honda *et al.*, 1988*a,b*). Moreover, both PTHrP RIA and bioassay confirmed the production of PTHrP in these tumour tissues. These data indicated a very close relationship between the synthesis of PTHrP-like peptides and the development of HHM in most cases of these transplantable tumours, supporting our previous findings with clinical samples (Tsuchihashi *et al.*, 1990).

Several published reports which examined bioactivity of PTH-like substances produced by HHM-associated tumours suggest a molecular size heterogeneity (Burtis *et al.*, 1987; Moseley *et al.*, 1987; Stewart *et al.*, 1987*a,b*; Strewler *et al.*, 1987). Current thinking is that these molecules are likely to be PTHrP. Gel filtration studies combined with RIA and bioassay on three tumour tissue extracts revealed at least two different molecules with PTHrP-like immunological and biological activity. These results indicated that PTHrP molecules with a molecular size equal to or greater than cytochrome C were the major components in these tumour extracts. Also, it is worth noting that each bioassayable peak contained IR-PTHrP. Since PTHrP fragments with deletions at the amino-terminal portion act as potent antagonists for bioactive PTHrP molecules (Nagasaki *et al.*, 1989), the biologically-inactive IR-PTHrP peak (KEsC-2) may be a PTHrP-like molecule missing its amino-terminal portion.

The fact that four out of five tumours associated with HHM could be maintained as cultured cell lines permitted analysis of the biochemical characteristics and the amount of PTHrP released into the culture media. A major form of immunoaffinity-purified IR-PTHrP released into culture media from KEsC-2 cells possessed the same molecular size as that detected in the tumour tissue extract; these findings suggested that molecules detected in the tumour tissue extracts were actively secreted and played a major role in inducing hypercalcaemia. The present study also demonstrated that four tumour cell lines associated with HHM secreted IR-PTHrP in amounts ranging from 3.0 to 450.0 pmol/1.5 × $10^9$ cells (approximately equivalent to 1.5 g wet weight) 24 $h^{-1}$. Using osmotic minipumps, we have reported that a subcutaneous infusion of 1,200 pmol 24 $h^{-1}$ of PTHrP(1–34) in nude mice induced a remarkable hypercalcaemia (average 17.7 ± 2.4 mg $dl^{-1}$; Nagasaki *et al.*, 1989). Recently, we found that a dose of 100 pmol 24 $h^{-1}$ also induced significant hypercalcaemia (average 10.0 ± 0.34 mg $dl^{-1}$) in the test animals compared to 8.3 ± 0.3 mg $dl^{-1}$ in the controls (Nagasaki, K. *et al.*, manuscript in preparation). Therefore, the amount of PTHrP secreted from KEsC-2 cells and presumably from FA-6 cells seems to be sufficient to induce hypercalcaemia. However, it is necessary to remember that tumour cell size and PTHrP-producing activity *in vitro* does not always correlate closely with cell size and activity *in vivo*. Thus, to substantiate this claim measurements of plasma PTHrP levels in the PTHrP-infused hypercalcaemic nude mice and in tumour-bearing hypercalcaemic animals are necessary.

Consideration of the two cell lines associated with hypercalcaemia, but producing rather small amounts of PTHrP

highlights the need to examine another possibility. SEKI, a melanoma cell line, produces large amounts of transforming growth factor (TGF)-α (Imanishi *et al.*, 1989). Since TGF-α possesses bone-resorbing activity (Tashjian *et al.*, 1986), this growth factor could aggravate PTHrP-induced hypercalcaemia in this case. There are a number of compounds with bone-resorbing activity including prostaglandins, TGF-β, epidermal growth factor, interleukin-1α and -1β (Bringhurst *et al.*, 1986; Tashjian *et al.*, 1986; Linkhart *et al.*, 1989; Sato *et al.*, 1987; Mundy, 1988). Examination of the production of these compounds in these transplantable tumours is necessary to better understand their potential roles in this morbidity in these animal models. Studies of plasma levels of these compounds, including PTHrP, in tumour-bearing nude mice is one approach to further our understanding.

This investigation was supported in part by a Research Grant from the Princess Takamatsu Cancer Research Fund, by a Grant-in-Aid from the Ministry of Health and Welfare for a Comprehensive 10-year Strategy of Cancer Control, by Grants-in-Aid for Cancer Research (62S-1, 1-5 and 1-33) from the Ministry of Health and Welfare, by the Special Coordination Funds from the Science and Technology Agency for Promoting Science and Technology and by a Grant-in-Aid from the Mochida Memorial Foundation for Medical and Pharmaceutical Research. Dr Miyake is a Research Resident Fellow of the Foundation for Promotion of Cancer Research, Japan. The authors thank Dr Y. Maeda (Tokyo Metropolitan Komagome Hospital, Tokyo, Japan) and Dr Y. Nagata (National Defence Medical College, Saitama, Japan) for providing cancer cell lines of KEsC-2 and FA-6, respectively. They also thank Ms K. Otsubo and Ms M. Ebinuma for their excellent technical assistance.

### References

BRINGHURST, F.R., BIERER, B.E., GODEAU, F., NEYHARD, N., VARNER, V. & SEGRE, G.V. (1986). Humoral hypercalcemia of malignancy: release of a prostaglandin-stimulating bone-resorbing factor in vitro by human transitional-cell carcinoma cells. *J. Clin. Invest.*, **77**, 456.

BUDAYR, A.A., NISSENSON, R.A., KLEIN, R.F. & 5 others (1989*a*). Increased serum levels of a parathyroid hormone-like protein in malignancy-associated hypercalcemia. *Ann. Intern. Med.*, **111**, 807.

BUDAYR, A.A., HALLORAN, B.P., KING, J.C., DIEP, D., NISSENSON, R.A. & STREWLER, G.J. (1989*b*). High levels of a parathyroid hormone-like protein in milk. *Proc. Natl Acad. Sci. USA*, **86**, 7183.

BURTIS, W.J., WU, T., BUNCH, C. & 5 others (1987). Identification of a novel 17,000-dalton parathyroid hormone-like adenylate cyclase-stimulating protein from a tumor associated with humoral hypercalcemia of malignancy. *J. Biol. Chem.*, **262**, 7151.

BURTIS, W.J., BRADY, T.G., ORLOFF, J.J. & 7 others (1990). Immunochemical characterization of circulating parathyroid hormone-related protein in patients with humoral hypercalcemia of cancer. *New Engl. J. Med.*, **322**, 1106.

GKONOS, P.J., HAYES, T., BURTIS, W. & 4 others (1984). Squamous carcinoma model of humoral hypercalcemia of malignancy. *Endocrinology*, **115**, 2384.

HONDA, S., YAMAGUCHI, K., SUZUKI, M. & 4 others (1988*a*). Expression of parathyroid hormone-related protein mRNA in tumors obtained from patients with humoral hypercalcemia of malignancy. *Jpn. J. Cancer Res. (Gann)*, **79**, 677.

HONDA, S., YAMAGUCHI, K., MIYAKE, Y. & 9 others (1988*b*). Production of parathyroid hormone-related protein in adult T-cell leukemia cells. *Jpn. J. Cancer Res. (Gann)*, **79**, 1264.

IKEDA, K., MATSUMOTO, T., FUKUMOTO, S. & 7 others (1988). A hypercalcemic nude rat model that completely mimics human syndrome of humoral hypercalcemia of malignancy. *Calcif. Tissue Int.*, **43**, 97.

IMANISHI, K., YAMAGUCHI, K., SUZUKI, M., HONDA, S., YANAIHARA, N. & ABE, K. (1989). Production of transforming growth factor-α in human tumour cell lines. *Br. J. Cancer*, **59**, 761.

KAMEYA, T., KODAMA, T. & SHIMOSATO, Y. (1982). Morphology of lung cancer in relation to its function. In *Morphogenesis of Lung Cancer*, Vol. 2, Shimosato, Y., Melamed, M.R. & Nettesheim, P. (eds) p. 107. CRC Press: Florida.

KUKREJA, S.C., SHEVRIN, D.H., WIMBISCUS, S.A. & 5 others (1988). Antibodies to parathyroid hormone-related protein lower serum calcium in athymic mouse models of malignancy-associated hypercalcemia due to human tumors. *J. Clin. Invest.*, **82**, 1798.

LINKHART, T.A., MOHAN, S., JENNINGS, J.C. & BAYLINK, D.J. (1989). Copurification of osteolytic and transforming growth factor β activities produced by human lung tumor cells associated with humoral hypercalcemia of malignancy. *Cancer Res.*, **49**, 271.

MANGIN, M., WEBB, A.C., DREYER, B.E. & 9 others (1988). Identification of a cDNA encoding a parathyroid hormone-like peptide from a human tumor associated with humoral hypercalcemia of malignancy. *Proc. Natl Acad. Sci. USA*, **85**, 597.

MARUNO, K., YAMAGUCHI, K., ABE, K. & 5 others (1989). Immunoreactive gastrin-releasing peptide as a specific tumor marker in patients with small cell lung carcinoma. *Cancer Res.*, **49**, 629.

MEHDIZADEH, S., ALAGHBAND-ZADEH, J., GUSTERSON, B., ARLOT, M., BARDBEER, J.N. & LOVERIDGE, N. (1989). Bone resorption and circulating PTH-like bioactivity in an animal model of hypercalcaemia of malignancy. *Biochem. Biophys. Res. Commun.*, **161**, 1166.

MOSELEY, J.M., KUBOTA, M., DIEFENBACH-JAGGER, H. & 8 others (1987). Parathyroid hormone-related protein purified from a human lung cancer cell line. *Proc. Natl Acad. Sci. USA*, **84**, 5048.

MUNDY, G.R. (1988). Hypercalcemia of malignancy revisited. *J. Clin. Invest.*, **82**, 1.

NAGASAKI, K., YAMAGUCHI, K., MIYAKE, Y. & 7 others (1989). *In vitro* and *in vivo* antagonists against parathyroid hormone-related protein. *Biochem. Biophys. Res. Commun.*, **158**, 1036.

NAGATA, N., AKATSU, T., KUGAI, N. & 6 others (1989). The tumor cells (FA-6) established from a pancreatic cancer associated with humoral hypercalcemia of malignancy: a simultaneous production of parathyroid hormone-like activity and transforming growth factor activity. *Endocrinol. Japon*, **36**, 75.

OVEJERA, A.A., HOUCHENS, D.P., BARKER, A.D. & VENDITTI, J.M. (1978). Inactivity of DL-amygdalin against human breast and colon tumor xenografts in athymic (nude) mice. *Cancer Treat. Rep.*, **62**, 576.

SATO, K., FUJII, Y., ONO, M., NOMURA, H. & SHIZUME, K. (1987). Production of interleukin 1α-like factor and colony-stimulating factor by a squamous cell carcinoma of the thyroid (T3M-5) derived from a patient with hypercalcemia and leukocytosis. *Cancer Res.*, **47**, 6474.

SHIMOYAMA, M. (1975). SEKI strain. In *In vitro Culture of Human Cancer Cells* (in Japanese), Oboshi, S. & Sugano, H. (eds) p. 208. Asakura Shoten: Tokyo, Japan.

STEWART, A.F., HORST, R., DEFTOS, L.J., CADMAN, E.C., LANG, R. & BROADUS, A.E. (1980). Biochemical evaluation of patients with cancer-associated Hypercalcemia: evidence for humoral and nonhumoral groups. *N. Engl. J. Med.*, **303**, 1377.

STEWART, A.F., INSOGNA, K.L., GOLTZMAN, D. & BROADUS, A.E. (1983). Identification of adenylate cyclase-stimulating activity and cytochemical glucose-6-phosphate dehydrogenase-stimulating activity in extracts of tumors from patients with humoral hypercalcemia of malignancy. *Proc. Natl Acad. Sci. USA*, **80**, 1454.

STEWART, A.F., WU, T., GOUMAS, D., BURTIS, W.J. & BROADUS, A.E. (1987*a*). N-terminal amino acid sequence of two novel tumor-derived adenylate cyclase-stimulating proteins: identification of parathyroid hormone-like and parathyroid hormone-unlike domains. *Biochem. Biophys. Res. Commun.*, **146**, 672.

STEWART, A.F., BURTIS, W.J.,. WU, T., GOUMAS, D. & BROADUS, A.E. (1987*b*). Two forms of parathyroid hormone-like adenylate cyclase-stimulating protein derived from tumors associated with humoral hypercalcemia of malignancy. *J. Bone Mineral Res.*, **2**, 587.

STREWLER, G.J., STERN, P.H., JACOBS, J.W. & 5 others (1987). Parathyroid hormonelike protein from human renal carcinoma cells: structural and functional homology with parathyroid hormone. *J. Clin. Invest.*, **80**, 1803.

SUVA, L.J., WINSLOW, G.A., WETTENHALL, R.E.H. & 10 others (1987). A parathyroid hormone-related protein implicated in malignant hypercalcemia: cloning and expression. *Science*, **237**, 893.

TASHJIAN, A.H., Jr, VOELKEL, E.F., LLOYD, W., DERYNCK, R., WINKLER, M.E. & LEVINE, L. (1986). Actions of growth factors on plasma calcium: epidermal growth factor and human transforming growth factor-alpha cause elevation of plasma calcium in mice. *J. Clin. Invest.*, **78**, 1405.

TSUCHIHASHI, T., YAMAGUCHI, K., MIYAKE, Y. & 9 others (1990). Parathyroid hormone-related protein in tumor tissues obtained from patients with humoral hypercalcemia of malignancy. *J. Natl Cancer Inst.*, **82**, 40.

WEIR, E.C., INSOGNA, K.L., BROWNSTEIN, D.G., BANDER, N.H. & BROADUS, A.E. (1988). In vitro adenylate cyclase-stimulating activity predicts the occurrence of humoral hypercalcemia of malignancy in nude mice. *J. Clin. Invest.*, **81**, 818.

YENDT, E.R. (1972). Disorders of calcium, phosphorus, and magnesium metabolism. In *Clinical Disorders of Fluid and Electrolyte Metabolism, 2nd Edition.* Maxwell, M.H. & Kleeman, C.R. (eds) p. 407. McGraw-Hill Book Co.: New York.